# Intervention program to modify the smoking habit in employee group of Athens' social welfare organization using motivational interviewing techniques and Trans theoretical Model of Behavior Change

**DOI:** 10.1186/1617-9625-12-S1-A36

**Published:** 2014-06-06

**Authors:** Eleftheria Kenanidou

**Affiliations:** 1 Health Visitor, Athens, Greece

## Background

Smoking is globally the most important risk factor for health and a major factor of mortality. It is responsible for many diseases, such as cardiovascular diseases, digestive system, musculoskeletal system, respiratory system, coronary artery disease, vascular strokes, while destroying the immune system and increases the risk of infections. The aim of this study was to assess the effectiveness of the motivational interviewing and Trans theoretical Model of Behavior Change related to the modification of the smoking habit.

## Materials and methods

The present study took place in Athens' Social Welfare Organization. The intervention program was held as part of a boarder program, whose purpose was to amend behaviors that constitute risk factors for health and related to the way of living and specifically with the smoking habit. Four Questionnaires were used for this study. One of these is a Health Questionnaire with questions about nutrition, physical activity, stress levels and behaviors related to smoking. Also, the Change Questionnaire about smoking, the Socrates 8D Questionnaire and finally a process evaluation Questionnaire. Ten (10) people were placed randomly in the intervention and control groups. People in intervention group participated in six (6) sessions lasting 35 minutes using the motivational interviewing techniques, while those in the control group received one meeting session lasting 25 minutes with information about the benefits of quitting smoking and the pharmacological treatment options.

## Results

While initially the changes in relation to the smoking habits ranged from mild to effective, in the intervention group all people after the end of the intervention program reported the desire of stop (60%) and reduce (40%) smoking. In both groups there is an upward trend in the intensity of thoughts, expectations and feelings associated with smoking. None of those two groups mentioned abstinence and quitting smoking.

## Conclusions

Also, the population sample is not representative of the general population and the results couldn't be generalized. Finally, the analysis of the results showed the positive attitude of all people in the implementation of such programs.

